# Interrater agreement for characterization of capitellar osteochondritis dissecans using photon-counting computed tomography technology

**DOI:** 10.1016/j.jseint.2026.101676

**Published:** 2026-02-28

**Authors:** Esli D. Steenbeek, Thom van der Laan, Ronald Booij, Sara J. Baart, Coen Otterspeer, Mariëlle E.F. Olsthoorn, Ilknur Sanli, Denise Eygendaal, Edwin H.G. Oei, Anna van der Windt

**Affiliations:** a Department of Orthopaedics and Sports Medicine, Erasmus MC University Medical Center Rotterdam, Rotterdam, The Netherlands; b Department of Radiology and Nuclear Medicine, Erasmus MC University Medical Center Rotterdam, Rotterdam, The Netherlands; c Department of Biostatistics, Erasmus MC University Medical Center Rotterdam, Rotterdam, The Netherlands

**Keywords:** Photon-counting, Computed tomography, Osteochondritis dissecans, Capitellum, Elbow, Interrater agreement, Intraclass correlation coefficient

## Abstract

**Background:**

Imaging in children with capitellar osteochondritis dissecans (COCD) is crucial for surgical decision-making but remains challenging. Novel photon-counting computed tomography (PCCT) technology enables superior resolution while reducing radiation dose. This study aims to describe interrater agreement (IRA) for COCD characteristics assessed with PCCT and to make a comparison with conventional CT.

**Methods:**

At a tertiary referral hospital, anonymized PCCT and conventional CT scans of COCD lesions were assessed systematically and independently by 2 experienced clinicians and 2 musculoskeletal radiologists, excluding postoperative scans. The intraclass correlation coefficient (ICC) with 95% confidence intervals was used to describe IRA among all raters.

**Results:**

In the PCCT group (n = 29), ICCs for loose body count, lesion size in the sagittal plane, presence of an empty defect, and presence of an osseous bridge were 0.75 (95% CI: 0.61–0.86), 0.68 (0.51–0.81), 0.64 (0.47–0.78), and 0.60 (0.43–0.76), respectively. ICCs of physeal status, lateral wall involvement, fragmentation, depth, and tilting had a lower bound of the 95% confidence interval below 0.4. In the conventional CT group (n = 12), ICCs for loose body count and osseous bridging were statistically significantly lower than in the PCCT group after adjustment for multiple testing (*P* = .009 and *P* = .025, respectively).

**Conclusion:**

PCCT assessment of COCD demonstrates at least substantial IRA for loose body count and at least moderate IRA for lesion size and the presence of an empty defect or bony bridge. Moreover, PCCT may enable higher IRA than conventional CT.

Capitellar osteochondritis dissecans (COCD) can put an end to participation in overhead sports and lead to irreversible osteoarthritic sequelae in pediatric athletes, if not treated in time.^7^ In early-stage COCD, the localized lesion can heal spontaneously with activity restrictions or immobilization.^21^^,^^22^ In the next stage of ongoing osteochondral segmentation, the prognosis is still good when the fragment is fixed timely within its origin.^20^ However, in end-stage COCD, fixation of loose bodies is no longer possible, and an empty defect remains with loss of the native cartilage.^20^

Whereas the treatment choice is primarily guided by imaging, reliable characterization of COCD lesions has turned out to be a challenge. Radiographs lack sensitivity,^11^^,^^16^ and whereas cross-sectional imaging provides more insight into the geometry of COCD lesions, 9 previously proposed magnetic resonance imaging (MRI) and CT classifications have shown fair interrater agreement (IRA) at best.^3^^,^^5^ As a result, the distinct description of key characteristics has been advocated.^3^^,^^5^ When describing COCD, conventional CT is more sensitive for fragmentation and secondary changes than MRI but exposes pediatric patients to high radiation dosages.^15^^,^^23^

Photon-counting computed tomography (PCCT) is a promising new technique that involves a fundamentally improved type of scanner with detectors that resolve the energy of each incoming X-ray photon. Benefits are superior spatial resolution, better intrinsic spectral imaging capabilities, reduced noise, and higher dose efficiency, as illustrated in Figure 1.^14^ Across various medical specialties, the number of studies on PCCT and its integration into clinical practice is rapidly increasing.^24^ However, to the authors' best knowledge, no literature exists about the utility of PCCT in COCD patients. It was hypothesized that PCCT is superior to conventional CT regarding key features of COCD that require high resolution, especially (ie, lesion size, loose body count, and the presence of a bony bridge between the progeny and parent bone).

**Figure 1:**
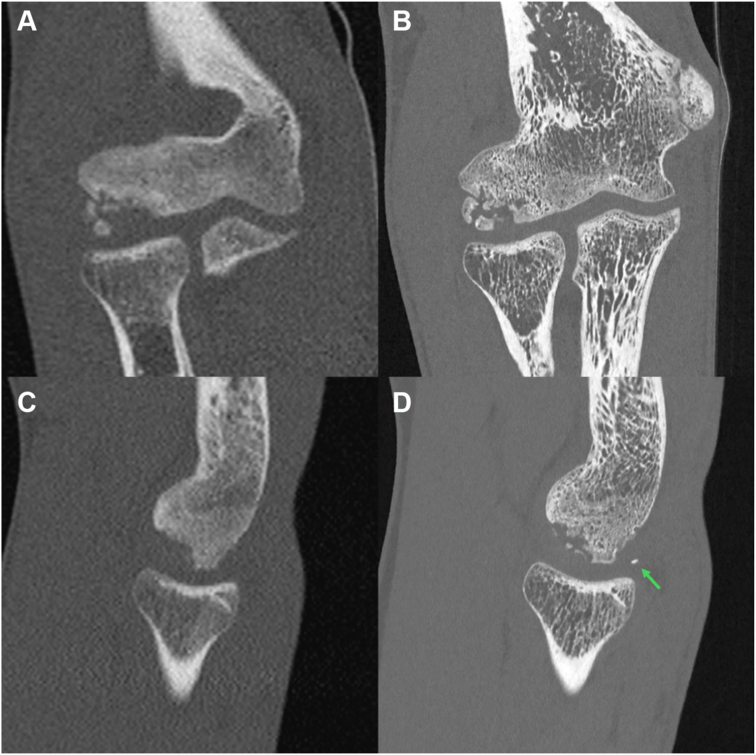
Conventional CT scan (**A** and **C)** versus photon-counting CT scan made 2 months later (**B** and **D)** of a patient with capitellar osteochondritis dissecans in the right elbow. An illustrative case of a 15-year-old male tennis player, training 5 times a week. For 2 years, he had pain in his lateral right elbow when playing (dominant side), and the pain was progressive for 3-4 months. The pain had an insidious onset without any causal moment, and there were no locking complaints. A conventional CT scan (A, coronal plane; C, sagittal plane) was made at a secondary referral hospital showing capitellar osteochondritis in the elbow joint. Two months later, the scan was repeated using photon-counting technology (B, coronal plane; D, sagittal plane) after referral to Erasmus MC, showing slightly increased fragmentation and increased dislocation of the fragments. Also, a loose body was detected (scan D, *green arrow*) in the radiocapitellar joint. This loose body was located in the ulnohumeral joint in scan C (not visible in this frame). Based on these scans, the patient went directly for arthroscopy, showing an unstable osteochondritis dissecans lesion. It was no longer possible to fixate fragments within their origin, so débridement and microfracturing were performed, as well as removal of the loose body. *CT*, computed tomography.

The primary aim of this explorative study is to describe IRA for a set of PCCT features currently used for surgical planning in COCD. The secondary aim is to compare IRA for COCD dimension in the sagittal plane, the number of loose bodies, and the presence of a bony bridge between PCCT and conventional CT.

## Methods

The 2011 Guideline for Reporting Reliability and Agreement Studies was followed.^12^

### Ethical considerations

All participants or parents/legal representatives (children <12 years old) or both (children 12 to 16 years old) signed an informed consent form for using their data for scientific research. The Erasmus Medical Center Medical Ethics Committee has declared the study exempt from review, given its retrospective nature (reference number MEC-2023-0663).

### Sampling method

Retrospectively, all patients with suspected COCD scheduled for PCCT between October 2022 and June 2024 at Erasmus MC University Medical Center Rotterdam (Erasmus MC), the Netherlands, were retrieved. Any available conventional CT scans made of these patients, either at Erasmus MC or before referral, were also retrieved from their electronic health records. In case of multiple scans of the same patient (either multiple scans of the same elbow at different time points or bilateral scans), each scan was included as an individual case. Scans of patients aged below 18 years old with a diagnosis of COCD at the time of imaging were included. Scans of patients with acute traumatic osteochondral lesions or surgery on the elbow before the scan were excluded. The diagnosis of COCD was made by one of the clinicians of the Erasmus Upper extremity Research, Education & Care for children and Adults team based upon clinical and radiological evaluation. If there was any doubt about the diagnosis, one internationally recognized expert in the field (D.E.) made the final decision. Patient demographics, their clinical characteristics, and information about surgery performed after the scan were retrieved from the electronic health records.

The following PCCT technology was used: NAEOTOM Alpha with Quantum HD with 120 × 0.2 mm acquisition. This type of scanner is commercially available. Reconstructions were performed with a slice thickness and increment of 0.2/0.1 mm with kernel Br89. The radiation dose for the PCCT (CTDI_vol_) ranged from 4.5 to 6.5 mGy. Conventional CT scans obtained from the electronic health records were acquired using an energy-integrating detector CT. Scans were performed with a tube voltage of either 100 kV with a tin filter (100Sn) or 120 kV. Slice thickness varied from 0.6 mm to 2.0 mm. The radiation dose for the energy-integrating detector CT (CTDI_vol_) ranged from 0.95 to 10.14 mGy.

### Rating process

A scoring form was designed in the electronic data capture system Castor EDC (Castor Corporation, Claymont, United States) to assess a set of 10 widely recognized clinically relevant CT features (see Appendix 1). Four experienced observers were chosen, 2 musculoskeletal radiologists, 1 orthopedic surgeon, and 1 nurse practitioner, all of whom treat patients at Erasmus MC, national center of expertise for COCD. Scans were uploaded anonymously to the imaging software Syngo.via (Siemens Healthineers Corporation, Forchheim, Germany). Observers prospectively scored all scans while they were blinded to any patient details.

### Statistics

Case characteristics were stratified by scanning modality (ie, PCCT versus conventional CT). Continuous data were summarized with the median and interquartile range because all continuous variables had non-normal data distributions. To facilitate between-variable comparisons, the intraclass correlation coefficient (ICC) was chosen as the only index of IRA for all types of variables (two-way random, single-measures, absolute agreement model). Although Cohen's kappa is more commonly used for binary variables, the ICC is mathematically related and can be used instead with a similar interpretation.^9^^,^^19^

Regarding the primary study aim in the PCCT group, the ICCs with 95% confidence intervals were described for all COCD characteristics in the assessment form. When interpreting the ICC, its 95% confidence intervals were used rather than the ICC itself.^4^^,^^26^ The following cutoff values for the interpretation were applied: <0, poor agreement; 0-0.20, slight agreement; 0.21-0.4, fair agreement; 0.41-0.60, moderate agreement; 0.61-0.8, substantial agreement; 0.81-1.00, almost perfect agreement.^13^ The sample size calculation was as follows. With 4 raters, an expected reliability of 0.7 (ie, substantial agreement), a minimum acceptable reliability of 0.4 (ie, the lower border of moderate agreement), a significance level of 0.05, and a power of 80%, the required sample size was 17 according to Walter's formula.^13^^,^^25^

Regarding the secondary study aim, ICC values for 3 a priori selected key characteristics were compared between PCCT and conventional CT scans with a 2-sample z-test for proportions (two-sided, significance level of 0.05): the dimension of the COCD in the sagittal plane, the number of loose bodies, and the presence of a bony bridge. Multiple testing for these 3 comparisons was classified as disjunction testing according to Rubin's classification,^18^ which means that either scanning modality was considered superior if the difference for at least one of these 3 features is statistically significant. Therefore, adjustment for multiple testing was required, and the method of Benjamini and Hochberg was chosen.^1^^,^^10^

### Supplemental analyses

For literature comparisons, IRA was calculated for the popular classifications of OCD by Ferkel and Sgaglione and by Clanton and DeLee.^6^^,^^8^ Also, the ICCs for all COCD characteristics were described in the conventional CT group. Furthermore, several subgroup analyses were performed: for the group of scans of patients with complaints for a maximum of 2 years, and for assessment by radiologists versus clinicians. Finally, the main analysis was repeated with different measures of IRA as a sensitivity analysis. For binomial variables, the Fleiss' multirater kappa was used, and for the one ordinal variable (ie, physeal status), the Kendall's coefficient of concordance was used.

## Results

A total of 49 scans of 36 children were retrieved, 6 of which were excluded because of earlier elbow arthroscopy in a secondary-level hospital and 2 of which were excluded because the lesion developed after major trauma to the elbow with a concomitant radial head fracture that required surgical fixation. Twenty-nine PCCT and 12 conventional CT scans of 32 patients were included in the analysis. From the 9 patients who contributed to multiple scans, 4 patients had bilateral scans, and 5 patients had the same side scanned twice, first by conventional CT and then by PCCT. At the time of the PCCT scan, patients had a median age of 14.1 (interquartile range, 13.4-15.6) years and were predominantly female gymnasts with their dominant side affected (see Table I). After the scan, arthroscopy was performed in two-thirds of cases.

**Table I:** Characteristics of the study population at the time of the scan∗.

Characteristic	PCCT (n = 29)	Conventional CT (n = 12)
Age in yr (median, IQR)	14.1 (13.4-15.6)	14.5 (13.1-15.7)
Female sex (%)	18 (62.1)	7 (58.3)
Right side (%)	23 (79.3)	10 (83.3)
Dominant side (%)	20 (76.9)	8 (88.9)
Most important UE sport (%)		
Gymnastics	10 (34.5)	3 (25.0)
Hockey	3 (10.3)	5 (41.7)
Fitness	3 (10.3)	0 (0.0)
Other†	12 (41.4)	4 (33.3)
None	1 (3.4)	0 (0.0)
Asymptomatic (%)	2 (6.9)	0 (0.0)
Duration of symptoms in mo (median, IQR)‡	12.2 (7.3-25.6)	13.2 (3.1-23.7)
Locking complaints (%)	14 (48.3)	6 (54.5)
Location scan (%)		
Secondary level hospital	0 (0.0)	8 (66.7)
Tertiary level hospital	29 (100.0)	4 (33.3)
Arthroscopy after scan (%)	19 (65.5)	7 (58.3)

*CT*, computed tomography; *PCCT*, photon-counting computed tomography; *IQR*, interquartile range; *UE*, upper extremity.

∗ Missing values PCCT versus conventional CT group: dominant side, 10.3 versus 25.0%; asymptomatic, 0 versus 8.3%; duration of symptoms, 3.7 versus 16.7%; locking complaints, 0 versus 8.3%.

† Ie, tennis, volleyball, kickboxing, basketball, BMX, free running, motor racing, soccer keeping, golf, street dancing, carpentry. The frequency of these sports was 2 or less per group.

‡ Excluding asymptomatic cases.

**Figure 2:**
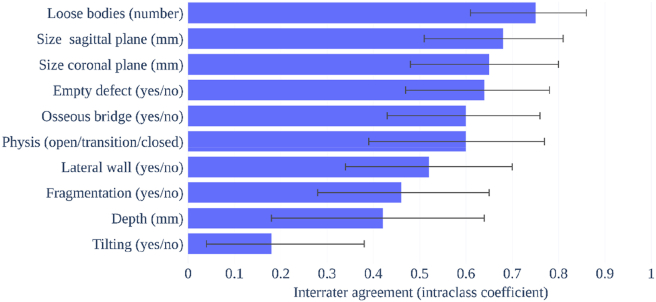
Descriptive analysis of interrater agreement for characterization of capitellar osteochondritis dissecans with photon-counting computed tomography with 95% confidence intervals (29 scans and 4 raters).

**Figure 3:**
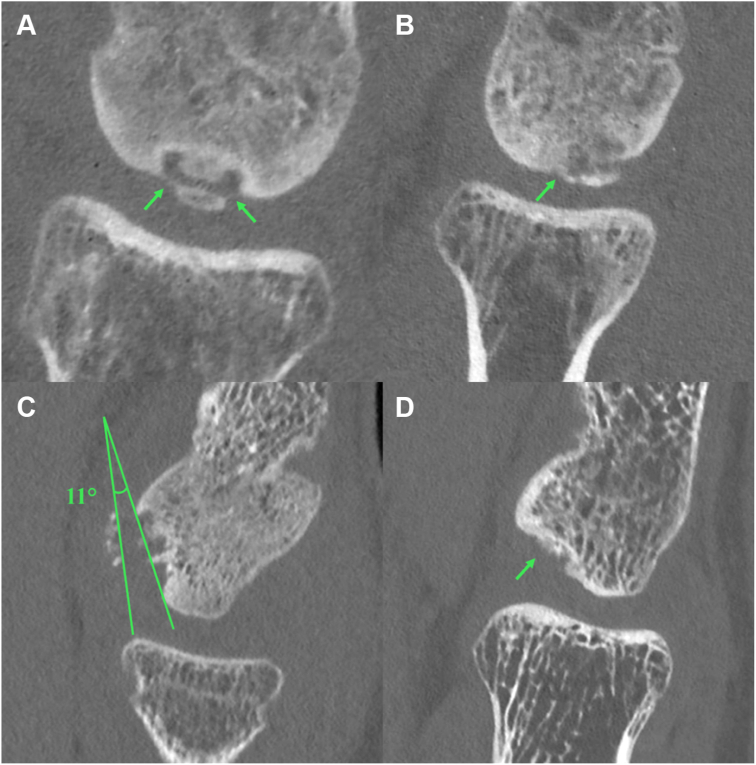
Photon-counting computed tomography images in sagittal plane of 4 different patients with capitellar osteochondritis dissecans (COCD) lesions, illustrative for the presence of an osseous bridge (**A** and **B)**, tilting (**C**), and an empty defect (**D**). These images show the complexity of describing COCD lesions. (**A**) The *green arrows* point toward 2 subtle structures that may be osseous bridges between the progeny and parent bone. The bone above in the picture is the humeral capitellum, and the bone below in the picture is the proximal radius. Four out of 4 raters scored “yes” for “a bony bridge between the COCD fragment and the bottom/edge of the defect.” (**B**) The *green arrow* points toward a connection between the progeny and parent bone that may be an osseous bridge. Three out of 4 raters scored “yes” for “a bony bridge between the COCD fragment and the bottom/edge of the defect.” (**C**) The angle measurement in *green* of 11° suggests tilting of the COCD fragment. Three out of 4 raters scored “yes” for “located within/in front of the defect and tilted relative to the joint line” (**D**) The *green arrow* points toward a defect in the capitellum. Four out of 4 raters scored “yes” for the presence of an “empty defect” in this scan.

In the descriptive analysis of the PCCT group (see Fig. 2), loose body count had the highest IRA of all characteristics (ie, substantial to almost perfect agreement), followed by lesion size in the sagittal plane (ie, moderate to almost perfect). Lesion size in the coronal plane, the presence of an empty defect, and the presence of a bony bridge had moderate to substantial agreement. Moreover, physeal status, lateral wall involvement, and presence of fragmentation demonstrated fair to substantial agreement, while the depth of the defect had poor to substantial agreement. The presence of tilting scored worst, with poor to fair IRA. Fig. 3 shows 4 slides from the assessed PCCT scans and illustrates what a bony bridge, fragment tilting, and an empty defect may look like.

Regarding the comparative analysis between the PCCT and CT groups (see Table II), IRA was statistically significantly higher in the PCCT group for the presence of a bony bridge and the number of loose bodies. There was no statistically significant between-group difference regarding lesion size in the sagittal plane.

**Table II:** Comparative analysis of interrater agreement for 3 key characteristics of capitellar osteochondritis dissecans with photon-counting computed tomography versus conventional computed tomography (41 scans, and 4 raters).

Characteristic	PCCT (n = 29)		Conventional CT (n = 12)		Between-group comparison	
	ICC∗	95% CI	ICC∗	95% CI	*P* value	Adj. *P* value
Bony bridge (yes/no)	0.60	0.43-0.76	0.19	−0.05-0.55	.017	.025
Loose bodies (number)	0.75	0.61-0.86	0.25	0.01-0.61	.003	.009
Size sagittal plane (mm)	0.68	0.51-0.81	0.61†	0.33-0.85†	.676	.676

*PCCT*, photon-counting computed tomography; *CT*, computed tomography; *ICC*, intraclass correlation coefficient; *CI*, confidence interval. *Adj*. = *P* value adjusted for multiple testing according to Benjamini-Hochberg.

∗ Two-way random, single-measures, absolute agreement model.

† One rater did not report any dimensions for 1 of the 12 scans and gave as reason that the scan was of too low quality to measure the size of the lesion.

The following findings in the supplemental analyses were noteworthy (see Supplementary Appendix 2). Both the Ferkel and Sgaglione and Clanton and DeLee classifications had poor IRA on PCCT (Supplementary Table S1). In the subgroup of patients with complaints ≤2 years, IRA was slightly decreased for some characteristics (Supplementary Table S3), and the differences between the PCCT and CT groups no longer reached statistical significance (Supplementary Table S4). On the contrary, in the subgroups of radiologists only and clinicians only, IRA increased (Supplementary Table S5). When comparing different measures of agreement, the Fleiss' multirater kappa showed similar results to the ICC (Supplementary Table S6), while the Kendall's coefficient of concordance was slightly higher than the ICC for physeal status (Supplementary Table S7).

## Discussion

In this IRA study, 2 experienced clinicians and 2 musculoskeletal radiologists systematically assessed 29 PCCT scans in children with COCD based on 10 features that determine surgical decision-making. Loose body count was the only feature with at least substantial IRA (ie, lower bound of the ICC 95% CI ≥ 0.61). Lesion size in the sagittal plane, lesion size in the coronal plane, presence of an empty defect, and presence of an osseous bridge had at least moderate IRA (ie, lower bound of the ICC 95% CI ≥ 0.41). In comparison, an additional set of 12 conventional CT scans was assessed, whereby these raters had considerably more disagreement regarding loose body count and the presence of an osseous bridge than in the PCCT group.

To the author's best knowledge, because of the novelty of PCCT technology, there is no previous literature about IRA of PCCT in COCD. However, regarding IRA of conventional CT, there are 2 previous studies. Claessen et al studied the IRA of 4 COCD classification systems and found poor to fair IRA for all of them.^5^ Our supplemental analysis confirms this finding since both classifications we studied showed poor IRA as well. Therefore, we advocate describing single features instead of using classifications. Furthermore, Bexkens et al studied lesion size and found an ICC of 0.93 (95% CI 0.86-0.97).^2^ The difference with our estimate may be explained by their use of 3-dimensional modeling and selection of cases requiring surgery only.

Strengths of this study include the large sample size of PCCT scans in a rare patient group and the addition of conventional CT scans as comparison. Also, assessment of the scans was blinded, so that assessors were not influenced by any clinical characteristics. Furthermore, scans were assessed independently and in a systematic manner with an a priori designed scoring form. The subgroup analyses provided an interesting hypothesis that IRA between radiologists and between clinicians is higher than between all raters combined. Finally, statistical comparisons were adjusted for multiple testing, decreasing the risk of type I errors.

This study also has limitations. More raters would have increased statistical precision, although the 95% confidence intervals in this study are reasonably small. The comparison between the PCCT and conventional CT group should also be interpreted with caution because of the small conventional CT sample size and the risk of selection bias. The PCCT and conventional CT scans were generally made of different patients, and in the 5 cases whereby the same patient contributed to both scanning modalities, the conventional CT scan was made earlier than the PCCT scan. Because of ethical considerations, one may not expose children to both PCCT and conventional CT at the same time. Nevertheless, patient characteristics were comparable between our PCCT and conventional CT groups.

In current clinical practice, access to PCCT is still limited because the transition from conventional CT to PCCT requires new hardware. However, future widespread adoption of PCCT is anticipated, driven by promising results across various medical specialties demonstrating improved image quality and diagnostic accuracy.^24^ In COCD specifically, PCCT is already the preferred imaging modality at our center because CT has higher sensitivity for secondary changes than MRI and because photon-counting technology minimizes radiation exposure in pediatric patients.^14^^,^^15^^,^^23^ In light of the current study, CT imaging of patients with COCD should preferably be performed with photon-counting technology if available. The actual clinical benefit of PCCT in COCD has yet to be elucidated.

Future studies should validate our findings. Moreover, we advocate the development of a core set of PCCT characteristics determining clinical decision-making. Previously published classifications for COCD correlate poorly with intraoperative findings,^17^ which is to be expected given their low IRA.^5^ We have now identified characteristics with high IRA (eg, loose body count, lesion size, and the presence of a bony bridge), which may therefore be more useful. These characteristics should be included in future diagnostic accuracy studies to determine clinical relevance, using intraoperative findings as the reference standard. Likewise, this study also identifies characteristics with low IRA (eg, fragment tilt), calling into question their use in future diagnostic accuracy studies and in current clinical decision-making.

## Conclusion

When interpreting PCCT scans of pediatric patients with COCD, evaluating the number of loose bodies demonstrates at least substantial IRA. Lesion size, the presence of an empty defect, and the presence of an osseous bridge show at least moderate IRA. In contrast, assessment of physeal status, lateral wall involvement, fragmentation, and depth may yield lower IRA, while the assessment of tilting is the most unreliable, demonstrating fair IRA at best. Furthermore, assessing the presence of an osseous bridge and loose body count may be more reliable with PCCT technology than with conventional CT scans. Both of these characteristics are key factors determining surgical decision-making.
