# Supplementary material for: Interrater agreement for characterization of capitellar osteochondritis dissecans using photon-counting computed tomography technology
Source: JSES Int. 2026 Feb 28;10(3):101676. doi: 10.1016/j.jseint.2026.101676 (PMC13091507; doi:10.1016/j.jseint.2026.101676)
Supplement: Appendix 1 [file mmc1.pdf]

Castor CDMS

PCCT-COCD study

Case Report Forms

Study ID	DCED41D1-06FB-4866-8C58-9187C54B83CD
Study Version	1.71
Generated at	22-09-2025 13:41:20
Generated by	
Participant ID	
Site	
Locked at	
Locked by	

Visits

Formulieren

Formulieren

1. Formulier

1.1 **How can this lesion be classified according to the Ferkel and Sgaglione classification? (see 1.2 for elucidation)***

- ◯ Stage I
- ◯ Stage IIA
- ◯ Stage IIB
- ◯ Stage III
- ◯ Stage IV

1.2 Ferkel and Sgaglioni classification

Imaging Type	Classification System	Criteria				
	Ferkel and Sgaglione[7]	**Stage I:** Cystic lesion present with an intact roof	**Stage IIA:** Cystic lesion with communication to the surface	**Stage IIB:** Open articular surface lesion with nondisplaced overlying fragment	**Stage III:** Nondisplaced fragment with lucency beneath lesion	**Stage IV:** Displaced fragment

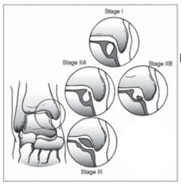

Example of Ferkel and Sgaglione stages in OCD of the talus

1.3 **How can this lesion be classified according to the Clanton en DeLee classification (zie 1.4 for elucidation)***

- ◯ Stage 1
- ◯ Stage 2
- ◯ Stage 3
- ◯ Stage 4

1.4 Clanton and DeLee classification

Imaging Type	Classification System	Criteria			
	Clanton and DeLee[6]	**Stage 1:** Depressed osteochondral fracture	**Stage 2:** Osteochondral fragment attached by an osseous bridge	**Stage 3:** Detached nondisplaced fragment	**Stage 4:** Displaced fragment

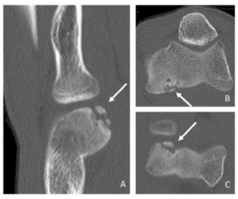

CT scan of OCD in the elbow. The arrow indicates OCD **stage 3** according to Clanton and DeLee.

What is the size of the COCD defect measured in coronal and sagittal plan and what is the depth of the defect (see 1.8 for elucidation)?

1.5 **In the coronal plane***

mm

1.6 **In the sagittal plane***

mm

1.7 **Depth (greatest measured)***

mm

1.8 Size and depth of the COCD defect

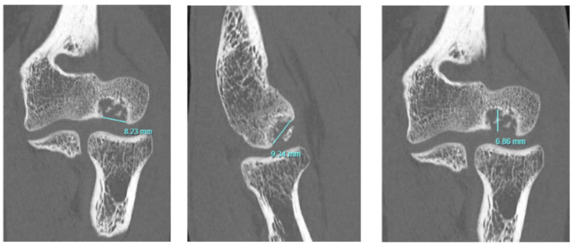

1.9 **Check the box if it concerns this lesion (see 1.10 for elucidation)***

- ☐ There is a bony connection (bridge) between the COCD fragment and the bottom/edge of the defect
- ☐ The COCD fragment is located within/in front of the defect and is fragmented (see 1.10 for elucidation)
- ☐ The COCD fragment is located within/in front of the defect and is tilted relative to the joint line
- ☐ There is an empty defect
- ☐ The COCD defect continues into the capitellar lateral wall (see 1.10 for elucidation)
- ☐ The growth plates are completely open
- ☐ The growth plates are partly closed
- ☐ The growth plates are closed

1.10 Characteristics of COCD

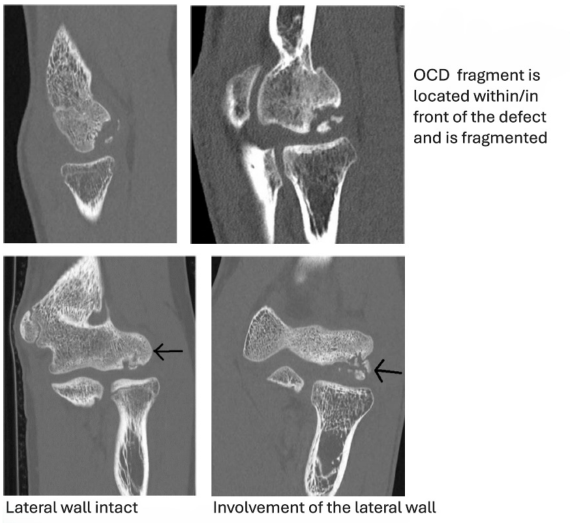

1.11 **How many loose bodies (located outside the COCD defect) are present on this scan?***

1.12 Comments (if any)
