# Supplementary material for: Interrater agreement for characterization of capitellar osteochondritis dissecans using photon-counting computed tomography technology
Source: JSES Int. 2026 Feb 28;10(3):101676. doi: 10.1016/j.jseint.2026.101676 (PMC13091507; doi:10.1016/j.jseint.2026.101676)
Supplement: Appendix 2 [file mmc2.pdf]

Appendix with supplementary material for:

Interrater agreement for characterization of capitellar osteochondritis dissecans using novel photon-counting computed tomography technology.

Contents:

Table S1. Descriptive analysis of interrater agreement for characterization of capitellar osteochondritis dissecans with photon-counting computed tomography, with addition of the Ferkel and Sgaglione and the Clanton and DeLee classifications (29 scans and 4 raters)

Table S2. Descriptive analysis of interrater agreement for characterization of capitellar osteochondritis dissecans with conventional computed tomography (12 scans and 4 raters)

Table S3. Descriptive analysis of interrater agreement for characterization of capitellar osteochondritis dissecans with photon-counting computed tomography: subgroup of patients with complaints for a maximum of two years (20 scans and 4 raters)

Table S4. Comparative analysis of interrater agreement for three key characteristics of capitellar osteochondritis dissecans with photon-counting computed tomography versus conventional computed tomography: subgroup of patients with complaints for a maximum of two years (29 scans and 4 raters)

Table S5. Descriptive analysis of interrater agreement for characterization of capitellar osteochondritis dissecans with photon-counting computed tomography: subgroup of radiologists and clinicians only (29 scans and 4 raters)

Table S6. Descriptive analysis of interrater agreement for characterization of capitellar osteochondritis dissecans with photon-counting computed tomography: Fleiss multirater kappa instead of intraclass coefficient (29 scans and 4 raters)

Table S7. Descriptive analysis of interrater agreement for characterization of capitellar osteochondritis dissecans with photon-counting computed tomography: Kendall's coefficient of concordance instead of intraclass coefficient (29 scans and 4 raters)

Table S1. Descriptive analysis of interrater agreement for characterization of capitellar osteochondritis dissecans with photon-counting computed tomography, with addition of the Ferkel and Sgaglione and the Clanton and DeLee classifications (29 scans and 4 raters)

	ICC*	95% CI
**Loose bodies (number)§**	0.75	0.61-0.86
**Size sagittal plane (mm)§**	0.68	0.51-0.81
**Size coronal plane (mm)§**	0.65	0.48-0.80
**Empty defect (yes/no)§**	0.64	0.47-0.78
**Bony bridge (yes/no)§**	0.60	0.43-0.76
**Physeal status (open/closed/trans.)§**	0.60	0.39-0.77
**Lateral wall involvement (yes/no)§**	0.52	0.34-0.70
**Fragmentation (yes/no)§**	0.46	0.28-0.65
**Depth (mm)§**	0.42	0.18-0.64
**Tilting (yes/no)§**	0.18	0.04-0.38
**Ferkel/Sgaglione (five categories)**	-0.00	-0.01-0.01
**Clanton/DeLee (four categories)**	-0.07	-0.16-0.07

Abbreviations: ICC = intraclass coefficient, CI = confidence interval, trans. = transition.
* Two-way random, single-measures, absolute agreement model.
§ As reported in main manuscript, see Figure 1.

Table S2. Descriptive analysis of interrater agreement for characterization of capitellar osteochondritis dissecans with conventional computed tomography (12 scans and 4 raters)

	ICC*	95% CI
**Lateral wall involvement (yes/no)**	1.00	n.a.
**Size coronal plane (mm)**	0.82§	0.62-0.94§
**Physeal status (open/closed/transition)**	0.70	0.39-0.89
**Size sagittal plane (mm)****	0.61§	0.33-0.85§
**Depth (mm)**	0.42	0.13-0.74
**Clanton/DeLee (four categories)**	0.34	0.09-0.67
**Empty defect (yes/no)**	0.34	0.06-0.68
**Ferkel/Sgaglione (five categories)**	0.28	0.04-0.62
**Fragmentation (yes/no)**	0.26	0.02-0.61
**Loose bodies (number)****	0.25	0.01-0.61
**Bony bridge (yes/no)****	0.19	-0.05-0.55
**Tilting (yes/no)**	0.16	-0.04-0.51

Abbreviations: ICC = intraclass coefficient, CI = confidence interval
* Two-way random, single-measures, absolute agreement model.
§ One rater did not report any dimensions for 1 of the 12 scans and gave as reason that the scan was of too low quality to measure the size of the lesion.
** As reported in main manuscript, see Table 2.

Table S3. Descriptive analysis of interrater agreement for characterization of capitellar osteochondritis dissecans with photon-counting computed tomography: subgroup of patients with complaints for a maximum of two years (20 scans and 4 raters)

	ICC§	95% CI
**Loose bodies (number)**	0.66	0.46-0.83
**Size sagittal plane (mm)**	0.60	0.39-0.79
**Size coronal plane (mm)**	0.66	0.46-0.83
**Empty defect (yes/no)**	0.54	0.32-0.75
**Bony bridge (yes/no)**	0.55	0.33-0.75
**Physeal status (open/closed/transition)**	0.51	0.26-0.73
**Lateral wall involvement (yes/no)**	0.64	0.44-0.81
**Fragmentation (yes/no)**	0.48	0.26-0.71
**Depth (mm)**	0.41	0.16-0.66
**Tilting (yes/no)**	0.21	0.04-0.46

Abbreviations: ICC = intraclass coefficient, CI = confidence interval
* A cut-off value of < 27 months was chosen to account for any logistic delay (maximum of 3 months at our hospital) between the outpatient clinic visit and the actual date of the scan.
§ Two-way random, single-measures, absolute agreement model.

Table S4. Comparative analysis of interrater agreement for three key characteristics of capitellar osteochondritis dissecans with photon-counting computed tomography versus conventional computed tomography: subgroup of patients with complaints for a maximum of two years (29 scans and 4 raters)

	PCCT (n = 20)		Conventional CT (n = 9)		Between-group comparison	
	ICC*	95% CI	ICC*	95% CI	P-value	Adj. p-value
**Bony bridge (yes/no)**	0.55	0.33-0.75	0.19	-0.09-0.63	0.071	0.213
**Loose bodies (number)**	0.66	0.46-0.83	0.35	0.06-0.73	0.119	0.179
**Size sagittal plane (mm)**	0.60	0.39-0.79	0.58	0.25-0.86	0.919	0.919

Abbreviations: PCCT = photon-counting computed tomography, CT = computed tomography, ICC = intraclass coefficient, CI = confidence interval, Adj. = p-value adjusted for multiple testing according to Benjamini-Hochberg
* Two-way random, single-measures, absolute agreement model.

Table S5. Descriptive analysis of interrater agreement for characterization of capitellar osteochondritis dissecans with photon-counting computed tomography: subgroup of radiologists and clinicians only

	Radiologists (n = 29, 2 raters)		Clinicians (n = 29, 2 raters)	
	ICC*	95% CI	ICC*	95% CI
**Loose bodies (number)**	0.81	0.63-0.90	0.86	0.72-0.93
**Size sagittal plane (mm)**	0.63	0.32-0.81	0.72	0.48-0.86
**Size coronal plane (mm)**	0.59	0.23-0.80	0.68	0.43-0.84
**Empty defect (yes/no)**	0.77	0.56-0.88	0.84	0.70-0.92
**Bony bridge (yes/no)**	0.59	0.28-0.78	0.62	0.34-0.80
**Physeal status (open/closed/transition)**	0.59	0.01-0.83	0.55	0.24-0.76
**Lateral wall involvement (yes/no)**	0.53	0.21-0.75	0.64	0.36-0.81
**Fragmentation (yes/no)**	0.60	0.31-0.79	0.48	0.15-0.71
**Depth (mm)**	0.55	0.20-0.77	0.44	-0.05-0.74
**Tilting (yes/no)**	0.09	-0.14-0.36	n.a. §	n.a. §

Abbreviations: ICC = intraclass coefficient, CI = confidence interval
* Two-way random, single-measures, absolute agreement model.
§ One rater reported 'no' for all scans regarding tilting. Therefore, no intraclass correlation coefficient could be calculated. The other rater reported 'yes' in 8 out of 20 scans.

Table S6. Descriptive analysis of interrater agreement for characterization of capitellar osteochondritis dissecans with photon-counting computed tomography: Fleiss multirater kappa instead of intraclass coefficient (29 scans and 4 raters)

	Fleiss multirater kappa	95% CI
**Empty defect (yes/no)**	0.62	0.48-0.77
**Bony bridge (yes/no)**	0.59	0.44-0.74
**Lateral wall involvement (yes/no)**	0.51	0.36-0.66
**Fragmentation (yes/no)**	0.45	0.30-0.60
**Tilting (yes/no)**	0.11	-0.03-0.26

Abbreviations: CI = confidence interval
* Two-way random, single-measures, absolute agreement model.

Table S7. Descriptive analysis of interrater agreement for characterization of capitellar osteochondritis dissecans with photon-counting computed tomography: Kendall's coefficient of concordance instead of intraclass coefficient (29 scans and 4 raters)

	KCC
**Physeal status (open/closed/transition)**	0.79

Abbreviations: KCC = Kendall's coefficient of concordance
